# Puberty and menstruation knowledge among young adolescents in low- and middle-income countries: a scoping review

**DOI:** 10.1007/s00038-019-01209-0

**Published:** 2019-02-10

**Authors:** Ernestina Coast, Samantha R. Lattof, Joe Strong

**Affiliations:** 1 Department of International Development, London School of Economics and Political Science, London, UK; 2 Department of Social Policy, London School of Economics and Political Science, London, UK

**Keywords:** Puberty, Menarche, Menstruation, Adolescence, Scoping review

## Abstract

**Objectives:**

This study presents a scoping review of evidence relating to knowledge and experiences of puberty and menstruation among females aged 10–14 years in low- and middle-income countries.

**Methods:**

Forty-four items from 12 countries were identified from a systematic scoping review and screening of 8083 items. Included studies were quality assessed.

**Results:**

A majority (40/44) of studies used school-based samples, and fifteen studies reported on interventions. Girls had inadequate knowledge about menstruation; menarche as a trigger for girls learning about menstruation was common. Adolescents struggled with menstrual hygiene. Negative emotions were associated with menarche and menstrual management. A minority of studies dealt explicitly with puberty. Most girls obtained information about menstruation and/or puberty from their mothers, although mothers were not necessarily girls’ preferred source for learning about these topics.

**Conclusions:**

Young adolescent girls are under-prepared for puberty and menstruation. Predominantly school-based studies mean we know little about young out-of-school adolescents. The evidence base lags behind the rise in interest from practitioners as well as the development (and evaluation) of puberty and/or menstruation interventions.

**Electronic supplementary material:**

The online version of this article (10.1007/s00038-019-01209-0) contains supplementary material, which is available to authorized users.

## Introduction

More than a quarter (26%) of the world’s population is female and of reproductive age, and most of them will menstruate monthly. Pre-menarche they will have begun puberty, involving physical, psychological, and cognitive transitions, lasting for most of the second decade of life. Puberty onset or menarche may define a time when girls’ roles change (Dolan et al. 2014; Mason et al. 2013; Mmari et al. 2016), including increased adult roles; changes in dress/deportment/behaviour; cessation or interruption of schooling; and mobility restrictions. How puberty is presented and experienced by girls and boys is different (Shaikh and Rahim 2006; Tasnim et al. 2009), with a “dominant narrative” of puberty as “shameful for girls while in contrast celebrating male virility” (UNESCO 2014, p. 11). The consequences of not knowing about puberty and menstruation include not understanding or being prepared for future fertility implications, for example. Among adolescent girls, fear and shame of menstruation are frequent themes that emerge both in country-specific studies and global reviews (Hennegan and Montgomery 2016; Mason et al. 2013; Sommer et al. 2015). The objective of this scoping review is to systematically map the range and quality of studies on knowledge and experiences of puberty and/or menstruation among young adolescents in low- and middle-income countries (LMICs).

In LMICs interventions and research tend to focus on older (15–19 years) adolescents (Igras et al. 2014). Early or young adolescence—10–14 years—incorporates the ages at which most girls begin puberty. Education about puberty is a “crucial” (UNESCO 2014) aspect of adolescent development. Recent guidance about puberty and menstruation education in LMICs (Haver and Long n.d.; House et al. 2012) has been accompanied by some countries developing national guidelines (MDWS 2015). These developments reflect growing understanding of the importance of puberty, and guidance documents include the need for information to be: age appropriate; culturally relevant; taught to both boys and girls; and deal with the software (e.g. knowledge) and hardware (e.g. absorbents, disposal) of menstruation.

Evidence documents the impact of menstrual hygiene management (MHM) on education, with problems of MHM affecting girls’ school attendance (WHO and UNICEF 2013). The management of puberty and menstruation might impact negatively on psychosocial well-being (e.g. stress, fear, embarrassment, shame). Parents may withdraw a daughter from school because puberty and menstruation are associated with reproduction and/or marriage, and/or concern about potential sexual advances by male students or teachers (Kirk and Sommer 2006).

Four systematic reviews reflect interest in menstruation as a research and/or intervention topic in LMICs; none focus exclusively on young adolescents or deal with puberty. Two reviews included menstruating females of all ages (Hennegan and Montgomery 2016; Sumpter and Torondel 2013), one included all female adolescents aged 10–19 (Chandra-Mouli and Patel 2017) and one included adolescent girls in India (van Eijk et al. 2016). Sumpter and Torondel’s systematic review focused on the health and social consequences of MHM (Sumpter and Torondel 2013). All four reviews focused on menarche and menstruation, separating it from broader pubertal transitions. The studies included in all four reviews were heterogeneous with associated risks of bias, and most included studies were deemed low quality across all four reviews. Chandra-Mouli and Patel noted varied design and context in their included studies, with inconsistencies in measuring key concepts (e.g. knowledge of menstruation), weaknesses around sample sizes, and high risks of bias in randomisation processes (Chandra-Mouli and Patel 2017). Inconsistently measured outcomes and an inability to draw synthesised conclusions were highlighted by two reviews (Hennegan and Montgomery 2016; van Eijk et al. 2016).

By situating menarche and menstruation within a broader pubertal transition (e.g. pubertal growth spurt, thelarche), our study extends beyond these reviews to incorporate young adolescent girls’ knowledge and experiences of puberty in LMICs. By combining puberty and menarche/menstruation, we draw attention to the ways in which two linked—but distinct—transitions and experiences are dealt with in the evidence base for young adolescents. By focusing on adolescents aged 10–14 years, our scoping review highlights the experiences of a critical age group that is likely to be experiencing, or about to experience, these transitions.

## Methods

**Fig. 1:**
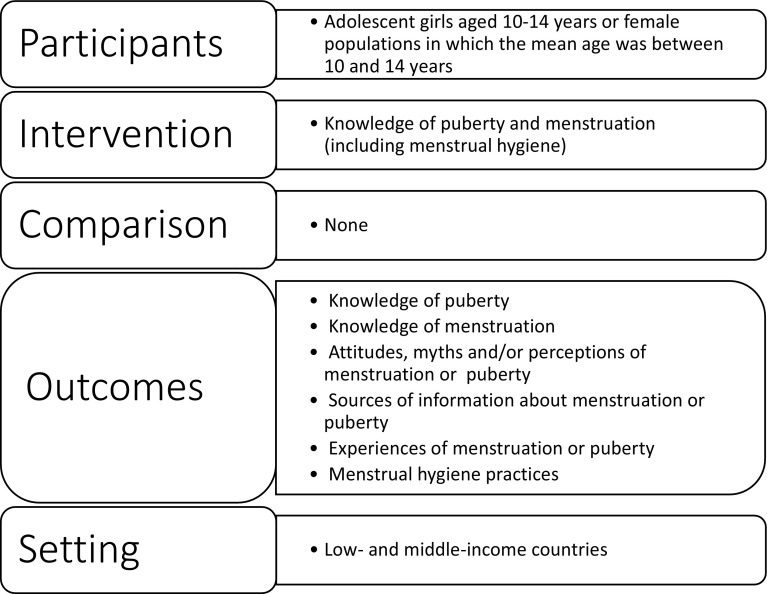
Populations, interventions, comparators, outcomes, and setting criteria for inclusion

We took a systematic, transparent, and reproducible approach to searching and including evidence in our scoping review in order to describe as widely as possible all of the relevant literature without, for example, limiting inclusion by study type or quality (Grant and Booth 2009; The Joanna Briggs Institute 2015). Our review, which used Arksey and O’Malley’s framework (Arksey and O’Malley 2005), focuses on evidence relating to early adolescent (aged 10–14 years) girls’ knowledge about and experiences of puberty and menstruation in LMICs (Fig. 1).

Studies on any population in LMICs were considered, using the World Bank Country and Lending Groups classification (World Bank 2016). Peer-reviewed journal articles and (non-peer-reviewed) grey literature, including reports, books or book chapters, whether in print or online, were eligible. For inclusion, studies must have:

- Included evidence relating to adolescent females aged 10–14 years. Studies including females outside this age range (e.g. ages 10–19 years) were included if the population’s mean age was between 10 and 14 years
- Stated as an outcome or aim to increase, or measured as an outcome, 10–14-year-old adolescent females’ knowledge of puberty or menstruation (including menstrual hygiene)
- Been published in English
- Been published between 1/1/2006 and 30/10/2017
- Measured at least one of these outcomes: knowledge of menstruation; menstrual hygiene practices; knowledge of puberty; attitudes, myths, and/or perceptions of menstruation/puberty; experiences of menstruation/puberty; or, sources of information about menstruation/puberty.

Quality was not a criterion for inclusion; we sought breadth and depth in our search (Khalil et al. 2016). Multiple references based on the same sample were not excluded in order to maximise the coverage of our review. By taking this approach—a variety of sources and a mixed body of evidence (type and methodology)—our scoping review generates an “overview of what is currently known and draws attention to areas where there are prominent knowledge gaps” (Davis et al. 2009, p. 1396). It establishes the extent of available evidence and how the research has been conducted (The Joanna Briggs Institute 2015).

Searches and application of inclusion criteria were conducted using an approach informed by the Preferred Reporting Items for Systematic Reviews and Meta-Analyses (PRISMA) flow approach (PRISMA 2017). Four sets of search terms (Table 1) were used in combination. Searches were not constrained by geographic location; studies focusing only on high-income countries were excluded at title and abstract (TIAB) screening. Search terms were tested in three databases (PubMed, ISI Web of Science (WoS), ScienceDirect) and crosschecked with medical subject headings dictionaries. Search term combinations were adapted to each database (e.g. wildcards, truncations). We systematically searched five electronic databases (PubMed, ISI WoS, ScienceDirect, JStor, Google Scholar) and one website (R4D).

**Table 1:** Search terms, their combinations, and database application

All 6 databases		All 6 databases		PubMed, ISI Web of Science, ScienceDirect		PubMed, ISI Web of Science, ScienceDirect
1. Adolescent terms	AND	2. Puberty and menstruation terms	AND	3. Knowledge and understanding terms	AND	4. Intervention/study type terms
adolescen*		puberty		know*		arrangement*
girl*		pubescen*		understand*		evaluat*
teenage*		sexual maturity		manage*		initiative*
youth*		catamenia		learn*		intervention*
pre-adolescen*		menstrua*		apprehen*		model*
		menarch*		comprehensi*		package*
		mense*		educat*		pilot*
				aware*		program*
				familiar*		project*
				proficien*		provision*
						regime*
						scheme*
						strateg*
						trial*
						approach*

*Refers to truncated word roots in order to capture multiple derivations, e.g. adolescen* will capture adolescent, adolescents, adolescence, etc

(adolescen* OR girl* OR teenage* OR youth* OR pre-adolescen*) AND (puberty OR pubescen* OR “sexual maturity” OR menstrua* OR menarch* OR mense* OR catamenia) AND (know* OR understand* OR manage* OR learn* OR apprehen* OR comprehensi* OR educat* OR aware* OR familiar* OR proficien*) AND (arrangement* OR evaluat* OR initiative* OR intervention* OR model* OR package* OR pilot* OR program* OR project* OR provision* OR regime* OR scheme* OR strateg* OR trial* OR approach*)

**Fig. 2:**
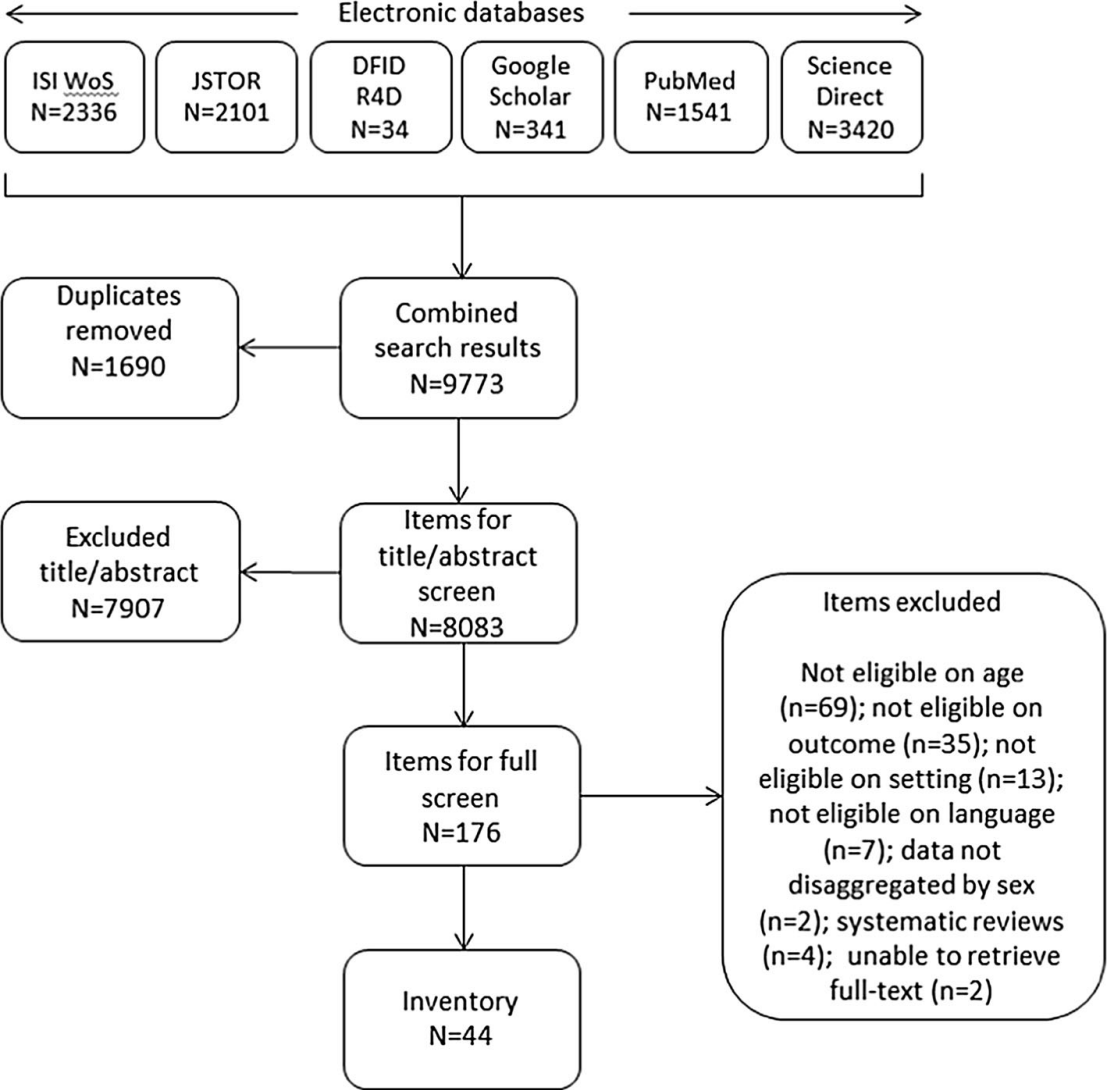
Search and screening results for ISI Web of Science (WoS), JSTOR, Department for International Development Research for Development (DFID R4D), Google Scholar, PubMed, Science Direct

This search generated 9773 items for screening. After duplicate removal, the 8083 remaining items were screened for inclusion on the basis of TIAB. We determined eligibility of all items, and unclear items were discussed. Where exclusion could not be determined on the basis of TIAB SRL and JS screened the full text. Decisions were made in favour of an inclusive approach where questions remained. Forty-four items were included in the scoping review. We were unable to retrieve two items for full-text screening (Bawono 2017; Hyang-Mi et al. 2006) (Fig. 2), as the items were unavailable from interlibrary loan or directly from the authors.

EC, SRL, and JS extracted data into SPSS for a randomly selected study to assure quality in data extraction. SRL and JS extracted data from all other studies. We present the results narratively, organised thematically. Included studies were quality assessed in duplicate [EC, SRL, and JS] using tools for quantitative and qualitative studies (EPHPP 2010; Solnes Miltenburg et al. 2013; Walsh and Downe 2006). The inclusion of quality assessment in a scoping review has been identified as useful for identifying gaps in the evidence base (Pham et al. 2014). As a scoping review, meta-analysis or meta-synthesis was inappropriate because of the heterogeneity of studies, populations, and outcomes.

Method limitations may have resulted in missed relevant items, specifically non-English items and those published outside of our time period. Since the searches were limited to six online sources, we may have missed relevant grey literature. The possibility is high that we missed some relevant in-service reports that were not intended or prepared for wider circulation. As an under-researched field, but one receiving growing attention, it is likely that evidence relating to young adolescents exists in the unpublished or grey literature (IRH 2011, 2013a, b, 2015a). As Sumpter et al. note, “there is a strong possibility that the best knowledge lies in the hands of those implementing programs” (Sumpter and Torondel 2013). Finally, despite attempts, we were unable to retrieve two items (Bawono 2017; Hyang-Mi et al. 2006) for screening that may have been eligible for inclusion.

## Results

Forty-four studies, from twelve countries, met all of the inclusion criteria (Supplementary Material, Table 1). The majority (30/44) were descriptive cross-sectional studies, with eight using a pre–post-test design and six randomised controlled trials (RCT). Most studies (40/44) used school-based samples (Table 2). Fifteen studies reported interventions ( Supplementary Material, Table 2), with interventions in Iran (*n* = 5) and India (*n* = 4) accounting for more than half of all intervention studies. The quality of included studies was weak (36/44); two studies were strong quality (Mason et al. 2013; Valizadeh et al. 2017) and six studies were of moderate quality (Tables 3, 4). We organise our results using the set of outcomes specified in our inclusion criteria, beginning with knowledge (and sources) of puberty and menstruation, then attitudes, myths, and/or perceptions before turning to experiences and practices.

**Table 2:** Characteristics of included studies

Country	India (17)
	Iran (7)
	Kenya (4)
	Nigeria (4)
	Bangladesh (3)
	Mexico (2)
	Nepal (2)
	Uganda (2)
	China (1)
	Jordan (1)
	Pakistan (1)
	Turkey (1)
Intervention	Yes (15)
Outcome measure	Knowledge of menstruation (25)
	Menstrual hygiene practices (22)
	Knowledge of puberty (9)
	Attitudes, myths, and/or perceptions about menstruation (16)
	Experiences of menstruation (26)/puberty (1)
	Sources of information about menstruation (17)/puberty (4)
Study type	Descriptive cross-sectional (quantitative) (23)
	Descriptive cross-sectional (qualitative) (5)
	Descriptive cross-sectional (mixed methods) (2)
	Pre–post-test (8)
	RCT (6)
Study population	In-school (40)
	In-school and out-of-school (1)
	Community-based sample (3)

**Table 3:** Summary of quality assessments for quantitative studies

References	Selection bias	Design	Confounders	Blinding	Data collection methods	Withdrawal/dropouts	Overall study quality
Adhikari et al. (2007)	***	***	-	**	***	***	Weak
Alam et al. (2017)	**	***	***	***	***	–	Weak
Anbumalar and Sasirekha (2016)	**	***	***	***	***	–	Weak
Afsari et al. (2017a)	**	*	*	**	**	*	Moderate
Afsari et al. (2017b)	**	***	***	***	**	–	Weak
Bosch et al. (2008)	***	***	-	**	***	**	Weak
Dabade and Dabade (2017)	**	***	***	***	***	–	Weak
Djalalinia et al. (2012)	***	*	***	**	*	*	Weak
Fazio et al. (2017)	**	***	***	**	**	***	Weak
Haque et al. (2014)	**	***	-	***	**	*	Weak
Hennegan et al. (2016)	**	*	***	***	***	***	Weak
Hossain et al. (2017)	**	***	***	***	***	–	Weak
Iliyasu et al. (2012)	*	***	-	**	***	*	Weak
Isguven et al. (2015)	**	***	-	**	***	*	Weak
Jena et al. (2017a)	**	***	***	***	***	–	Weak
Jena et al. (2017b)	**	***	***	**	***	*	Weak
Kapadia-Kundu et al. (2014)	**	*	*	**	***	*	Moderate
Kheirollahi et al. (2017)	**	*	***	**	*	***	Weak
Kumar et al. (2016)	**	***	***	***	***	–	Weak
Marván and Molina-Abolnik (2012)	**	***	-	**	***	*	Weak
Marván and Alcalá-Herrera (2014)	**	***	-	**	***	*	Weak
Mishra et al. (2017)	**	***	***	**	***	*	Weak
Montgomery et al. (2016)	**	*	*	***	**	***	Weak
Moodi et al. (2013)	***	**	*	***	**	*	Weak
Mumtaz and Ansari (2016)	**	*	***	**	**	**	Moderate
Nagaraj and Konapur (2016)	**	***	***	***	**	–	Weak
Phillips-Howard et al. (2016)	**	*	***	**	**	*	Moderate
Pratibha et al. (2016)	**	***	***	**	***	*	Weak
Ramachandra et al. (2016)	**	***	***	**	***	–	Weak
Saghi et al. (2016)	**	*	***	**	*	***	Weak
Shah et al. (2013)	**	***	-	**	***	*	Weak
Sharma et al. (2015)	***	**	***	**	*	*	Weak
Sharma et al. (2016)	**	***	***	**	***	–	Weak
Sharma and Moktan (2017)	**	***	***	**	**	–	Weak
Shoor (2017)	**	***	***	***	***	–	Weak
Sridhar and Gauthami (2017)	**	***	***	**	***	-	Weak
Su and Lindell (2016)	**	***	***	**	*	**	Weak
Um et al. (2010)	**	***	-	**	***	*	Weak
Valizadeh et al. (2017)	*	*	*	*	**	*	Strong

Key [scores based on EPHPP (2010)]

- Unclear/not specified

– Not relevant to article

*Strong

**Moderate

***Weak

**Table 4:** Summary of quality assessment for qualitative studies

References	Scope and purpose	Design	Sampling strategy	Analysis	Interpretation	Reflexivity	Overall
Agofure and Iyama (2016)	**	***	**	***	***	***	Moderate
Al Omari et al. (2016)	**	**	*	*	**	***	Weak
Bello et al. (2017)	**	*	*	*	*	***	Moderate
Chothe et al. (2014)	**	*	***	**	***	**	Weak
Mason et al. (2013)	*	*	*	*	*	**	Strong

Key [Based on Walsh and Downe (2006)]

*Strong

**Moderate

***Weak

### Knowledge of puberty

Few (9/44) studies address knowledge of puberty, and none include early sexual feelings, sexual development, sexuality, or related topics. These findings may reflect an elision by researchers and/or respondents of puberty with menstruation for girls. However, puberty is a broad set of changes, of which menstruation is one. One study, from Turkey, provided unambiguous definition of what the authors defined as “normal” puberty development (Isguven et al. 2015). In this survey of schoolgirls, three quarters (75.2%) self-identified as being knowledgeable about puberty and answered detailed questions about the first symptom of puberty (breast development, pubic and axillary hair, acne).

A study from Nigeria reported attitudes of mothers towards teaching their daughters about a variety of sexual and reproductive health issues. Most mothers (88.6%) agreed that adolescent girls should be informed about body changed at puberty (Iliyasu et al. 2012). In mothers’ narratives about discussions with their daughters, issues of puberty were bound up with issues relating to appearance, dress, use of cosmetics, courtship, sexuality and sexual behaviour. A comparative study of Nigeria and Kenya found that female adolescents were more likely to mention breast development than menstruation as associated with puberty. Adolescents went to great lengths to try to hide their bodily changes from others, including parents (Bello et al. 2017).

An Iranian study evaluated an educational program about puberty health on schoolgirls’ knowledge (Moodi et al. 2013). The study reported a significant difference between pre- and post-intervention results (*p* < 0.001), although there is little detail about either the intervention content or how knowledge scores were developed. The authors noted that due to shame and modesty, pre-pubertal girls tended not to discuss puberty with their mother. Though multiple studies from Iran focused on “puberty health” in their aims, the content frequently reported girls’ knowledge and experience of menstruation (Afsari et al. 2017a; Kheirollahi et al. 2017; Saghi et al. 2016; Valizadeh et al. 2017). This implies a potential elision by researchers of puberty with menstruation.

### Knowledge of menstruation

Knowledge of menstruation was a frequently included outcome (25/44). Across studies, girls have inadequate knowledge about menstruation and low levels of knowledge pre-menarche. A study from Bangladesh reported that 64% of girls were “reaching menarche in fear” (Bosch et al. 2008). In India, 60.3% girls did not know about menstruation prior to menarche, and they reported a poor understanding of the source and pathway of menstrual blood (Shah et al. 2013). Menarche as a trigger for girls learning about menstruation was common (Iliyasu et al. 2012; Mason et al. 2013).

Some studies framed questions in terms of “normal” age at menarche, "normal" duration of menstrual cycle, or "normal" flow. With one exception (Isguven et al. 2015), however, it is unclear how researchers define “normal”. Some studies did not report whether the response categories in surveys were closed or open ended (with post hoc coding). In Nepal, responses to the question “what is menstruation?” were reported using three categories (physiological, pathological, curse); it is unclear whether these were closed response categories, and if they were, how respondents understood the meanings (Adhikari et al. 2007).

Whilst most studies used survey questions, a study from India conducted a content analysis of questions about menstruation posed by girls aged 9–13 years, such as “Do we become infertile if our sanitary pad that is left in open is eaten or sniffed by a snake?” (Chothe et al. 2014). These questions provided culturally and contextually relevant insights into young adolescents’ understandings of menstruation. However, teachers were present during these question-and-answer sessions, potentially influencing girls’ questions.

Three studies examined interventions addressing knowledge of menstruation, all using a pre–post-test design (Haque et al. 2014; Shah et al. 2013; Sharma et al. 2015). An intervention in Bangladesh involved training schoolgirls on menstrual hygiene and reported significant (*p* < 0.001) impacts on some aspects of knowledge (e.g. menstruation blood is impure) but not others (e.g. cause of menstruation, origin of menstrual blood). This intervention study reported that recruiting female research assistants helped participants feel more comfortable discussing menstruation (Haque et al. 2014). Levels of knowledge about the biological (as opposed to hygiene) content of the intervention were not significantly improved. The authors posed a questionnaire during group discussions, raising issues about priming effects.

### Attitudes, myths, and/or perceptions about menstruation

Sixteen studies addressed fear, shame, secrecy, sexual vulnerability, positive/negative attitudes, and sociocultural constraints rooted in myths and taboos. Kenyan girls perceived pubescent, menstruating girls to be increasingly vulnerable to marriage, sexual advances, and abuse (Mason et al. 2013). A study in Bangladesh reported that 17% of girls asked questions about menstruation that researchers classified as myths and taboos (Bosch et al. 2008). Comparative analysis from India showed higher negative attitudes towards menarche in rural compared to urban areas (Anbumalar and Sasirekha 2016). A phenomenological analysis from Jordan found that adolescent girls considered talking about menarche to be “socially unacceptable” and “rude” (Al Omari et al. 2016).

Two studies measured girls’ attitudes towards menstruation in Bangladesh and Mexico. Upon reaching menarche in Bangladesh, nearly two-thirds (64%) of girls reported feeling scared (Bosch et al. 2008). Mexican schoolgirls were significantly (*p* < 0.0001) more likely to have negative feelings or feelings of secrecy about menstruation than they were to have positive feelings. Girls who were more knowledgeable and felt prepared for menstruation felt less negative and secretive (Marván and Molina-Abolnik 2012). A software intervention in China reported a statistically significant decrease in attitudes that menstruation was “debilitating and bothersome” in the group that received an educational intervention (Su and Lindell 2016).

### Sources of information about menstruation/puberty

Most studies revealed that girls obtained information about menstruation and/or puberty from their mothers. Mothers, however, were not necessarily girls’ preferred source of information. Some studies asked adolescents whom they thought should be teaching them. In Nepal, most girls (65.3%) preferred learning from a course book (Adhikari et al. 2007). In Turkey, when schoolgirls were asked who should provide education about puberty, the majority reported health professionals (54.4%) compared to families (30.0%) or teachers (5.9%) (2013). Whilst girls most often received information from females (e.g. mothers, sisters, friends, and female teachers), a few reported receiving information about menstruation and/or puberty from males (e.g. fathers, uncles, male teachers) (Isguven et al. 2015; Mason et al. 2013).

In some studies, it was unclear on what basis adolescents made judgements about the quality of menstruation information. A study from Nepal that asked schoolgirls whether they felt that they had been properly taught about menstruation reported that 98.0% of girls felt that they were not properly taught; it is unclear how “properly” was defined by the researchers or understood by the respondents (Adhikari et al. 2007).

### Experiences of menstruation or puberty

The majority of studies (26/44) included at least one outcome measure related to experience of menstruation; experience of puberty was rarely studied (1/44). Dependent upon the sociocultural context, some surveys asked questions about what girls can and cannot do whilst menstruating. A study from Nepal found that 70.7% of girls reported that girls cannot go to school and that 100% reported that girls cannot cook whilst menstruating (Adhikari et al. 2007).

Affective aspects of menarche and menstruation were reported. A study from Mexico that grouped girls by age at menarche found significant differences in the reporting of emotional responses to menarche. Compared to early maturers, late maturers were significantly less likely to report being scared, sad, and worried about menarche, and significantly more likely to report being excited or happy (Marván and Alcalá-Herrera 2014). Late maturers were significantly less likely to report that they would have to keep their first period a secret. The authors describe earlier maturers as experiencing a “truncated preparation time to develop the resources and skills needed to cope [with menarche]” (Marván and Alcalá-Herrera 2014).

Negative emotions related to menstruation were associated with issues of menstrual management, particularly around schooling. In some contexts, these emotions were linked to sociocultural norms around menstrual blood. In a study from Kenya it was reported, “Blood is something so secret that it is not recommended anyone to see” (Mason et al. 2013). A menstrual education programme in Bangladesh included a component on “hot or cold food affecting the menstrual cycle”. The post-test found the proportion of girls reporting that food temperature did not affect menses increased significantly (*p* = 0.001) (Haque et al. 2014). An intervention in Iran found significant differences in the reporting of emotions (confusing/scared/uncomfortable/good) about menarche between control and intervention groups (Djalalinia et al. 2012).

### Menstrual hygiene management

Our review includes studies with a wide range of definitions of “good” or “bad” MHM; in some studies any definition of “good” or “bad” was absent. How questions and response categories were phrased raises questions about the ways in which puberty and menstruation research is framed and understood by researchers. Adolescents reported struggling with menstrual hygiene, including obtaining resources for bathing/washing and absorbents. Kenyan schoolgirls said that competing for scarce resources (e.g. soap, water) could cause familial conflict and shame for girls unable to conceal menstruation (Mason et al. 2013). In India, girls hid used cloths in damp and dusty places (Shah et al. 2013).

Some studies considered sanitary pads to be any commercially branded or cloth pad, or grouped together sanitary pads and cotton in the analyses (Adhikari et al. 2007; Djalalinia et al. 2012). Other studies separated sanitary pads from cloth pads and reported on separate absorbents (Haque et al. 2014; Shah et al. 2013; Um et al. 2010). Most studies considered new cloth or specialty cloth to be hygienic.

Use of sanitary pads was generally low among studies that separated out sanitary pads from other types of absorbents (e.g. cloths), apart from a Nigerian study that reported 93.8% of adolescent schoolgirls used sanitary pads (Um et al. 2010). Whilst most girls preferred and valued sanitary pads, the cost made them unaffordable for many (Mason et al. 2013; Shah et al. 2013; Um et al. 2010). Girls rationed pads or used alternative absorbents (Mason et al. 2013). Evidence from India indicates that girls associated alternative and/or reused absorbents with illness and injury (Shah et al. 2013). One study from Kenya discussed how some girls reported obtaining money for sanitary pads in exchange for sex (Mason et al. 2013).

Studies evaluating interventions relating to MHM included both hardware (Phillips-Howard et al. 2016; Shah et al. 2013) and software interventions (Afsari et al. 2017a; Djalalinia et al. 2012; Haque et al. 2014; Kapadia-Kundu et al. 2014; Kheirollahi et al. 2017; Moodi et al. 2013; Nagaraj and Konapur 2016; Sharma et al. 2015; Su and Lindell 2016). Two linked studies combined hardware and software interventions (Hennegan et al. 2016; Montgomery et al. 2016), and assessed the association with school attendance; there was no association between reusable pads and puberty education and school attendance (Hennegan et al. 2016). Both education and provision of reusable sanitary pads were equally as effective in improving school attendance (Montgomery et al. 2016). A hardware intervention in rural India introduced *falalin* cloths, a low-cost, easily available red material with good absorption capacity. Girls using *falalin* cloths reported the fewest adverse quality-of-life issues (e.g. absent from school, skin abrasions), whereas girls using old cloths reported the most. At the study's end, 68% of adolescent girls preferred *falalin* cloths, whilst 32% of girls preferred sanitary pads (Shah et al. 2013); none preferred old cloths.

Haque et al. (Haque et al. 2014) delivered culturally adapted menstrual education, including menstrual hygiene demonstrations, to schoolgirls. They reported significant (p < 0.05) improvement in use of sanitary pads, the frequency of changing pads/cloths, drying the absorbent outside in sunlight, disposal of the absorbent by burial/burning/binning, and cleaning genitalia (Haque et al. 2014). In Iran, researchers randomly allocated students to one of three groups (control, trained by parents, trained by school) for a training programme. The trained groups bathed more frequently during menstruation than the control group and were more likely to use sanitary pads or cotton as a menstrual absorbent. This community-based educational intervention reported benefiting from including parents and teachers, although the study reports many limitations, including missing data values (Djalalinia et al. 2012). The education program implemented by Sharma et al. (2015) among Indian schoolgirls found a significant (*p* = 0.01) impact on the frequency of changing sanitary pads, genital cleaning, and reuse of cloths after washing. However, the study failed to include significant details about the study design, intervention, and outcomes.

## Discussion

Early adolescence represents a critical transitional period when gender norms can act in multiple ways to impact on adolescents’ lives (Igras et al. 2014). An over-arching theme emerges from our review: young adolescent girls in LMICs are under-prepared for puberty and menstruation.

Our review shows that puberty and menarche are inadequately researched and understood, in particular evidence relating to the experiences of young adolescents. The evidence base lags behind the rise in interest from practitioners in education and/or health (Adams et al. 2009; Haver and Long n.d.; House et al. 2012; UNESCO 2014) as well as the development (and evaluation) of interventions dealing with puberty and menstruation and its management such as Growing Up Smart (IRH 2015a; Rwanda: IRH 2015b); CycleSmart (Guatemala and Rwanda: IRH 2013a, b) and Choices (Nepal: IRH 2011). The volume of evidence is out of step with the scale of the issues associated with puberty, menstruation and its management.

The paucity of evidence on younger adolescents reflects, in part, the difficulties of researching this group. Many nationally representative surveys (e.g. DHS) include unmarried 15–19-year-olds in their sample; evidence is much less routinely collected from 10 to 14-year-olds. Evidence gaps may also reflect sociocultural contexts in which research with pre-menarcheal girls is constrained, especially across generations. Qualitative evidence underscores the proscribed nature of talking about puberty and menstruation, even among close relatives or friends. Whilst mothers were the main source of information reported in our scoping review, mothers themselves may lack sufficient, accurate knowledge of puberty or menstruation.

The most common interventions were software interventions that introduced or changed girls’ education about puberty and/or menstruation. The quality of intervention studies was low, with several studies providing insufficient detail on study design, intervention, evaluation, and results, thus hindering efforts to draw firm conclusions and replicate the interventions. No studies examined the impact of intervention duration or dosage. Evidence about sociocultural norms, including restrictions, relating to menstruation is critical to inform the design and content of appropriate interventions (House et al. 2012).

Some studies included evidence from people other than adolescent girls (e.g. teachers, mothers, fathers); adolescent boys were rarely included. The roles (colleague, peer, brother, father) played by men and boys in supporting girls and women in their MHM remain under-appreciated (House et al. 2012). Studies that also included adolescent boys usually did not ask them questions about menstruation; these questions were often only asked of girls.

Currently, the volume and quality of evidence does not align with the scale of the issues associated with puberty, menstruation and its management. Our scoping review suggests multiple agendas for future evidence and research, all framed by a need for evidence that is socioculturally and contextually relevant. The high proportion of school-based studies means we know very little about young out-of-school adolescents; some girls might be out of school for menstruation-related reasons. Evidence from non-school sample recruitment, including purposive recruitment of out-of-school adolescent girls, is needed. Better understanding the roles played by others—adolescent boys, parents, peers, teachers—and their understandings of puberty and menstruation would better contextualise girls’ experiences. Research would benefit from the production and use of consistent standards, grounded in evidence, of what is “acceptable” or “appropriate” knowledge. A majority of evidence does not specify or define key concepts related to knowledge and experience of puberty and/or menstruation. We still know very little about how adolescent girls implement MHM practices. In LMICs, effective MHM may require girls to compete for scarce resources (e.g. soap, water, money for proper absorbents), and factors such as socioeconomic status are likely to influence girls’ agency. Pubertal and menstrual stigma—their construction and consequences across girls’ lives (e.g. education)—remain poorly understood, as do the interventions to overcome them. Little is known about the impact of timing of puberty or menstruation interventions and education. There have been few attempts to directly compare “hardware” and “software” interventions, meaning that we do not know whether and how providing girls with physical supplies (e.g. pads) versus information (e.g. how to manage menstruation) leads to better outcomes. Finally, girls’ views and voices are rare in the current evidence base. Future research needs to facilitate girls’ meaningful participation in setting agendas and shaping interventions in order to capture the complexity of girls’ experiences.

## Electronic supplementary material

Below is the link to the electronic supplementary material.

Supplementary material 1 (DOCX 153 kb)
