# Supplementary material for: Puberty and menstruation knowledge among young adolescents in low- and middle-income countries: a scoping review
Source: Int J Public Health. 2019 Feb 10;64(2):293–304. doi: 10.1007/s00038-019-01209-0 (PMC6439145; doi:10.1007/s00038-019-01209-0)
Supplement: Supplementary file 1 — Supplementary material 1 (DOCX 153 kb) [file 38_2019_1209_MOESM1_ESM.docx]

Puberty and menstruation knowledge among young adolescents in low- and middle-income countries: a scoping review

International Journal of Public Health

Ernestina Coast, Samantha R. Lattof, Joe Strong

Electronic Supplementary Material

1. Summary table of included studies, including aims/objectives, population, study type, summary of main findings, and study quality (n=44)

2. Summary table of studies that included an intervention, including description of the intervention, evaluation, intervention claims, and reported limitations (n=15)

References

1. Summary table of included studies, including aims/objectives, population, study type, summary of main findings, and study quality (n=44)

| **Author, year [country]** | **Aim/objective(s)** | **Population** | **Study type** | **Summary of main findings by outcome** | **Study quality** |
| --- | --- | --- | --- | --- | --- |
| (Adhikari, Kadel et al. 2007)  [Nepal] | To evaluate the knowledge and practice on different aspects of menstrual hygiene in Nepal | 150 adolescent girls aged 13-15 years from 3 schools in rural Chitwan district | Descriptive cross-sectional | Knowledge of menstruation:  Although girls' knowledge of menstruation (40.6%) was better than their menstrual hygiene practice (12.9%), neither was satisfactory.  Menstrual hygiene practices:  Less than one third (29.3%) of girls changed their pad daily.  Experiences of menstruation:  The majority (96.7%) of girls felt emotions of upset or tension at their first menstruation.  Sources of information about menstruation:  The majority of girls (98.0%) believe that they were not properly taught about menstruation. | Weak |
| (Afsari, Mirghafourvand et al. 2017) [Iran] | To compare the effect of educating mothers and girls on attitudes towards puberty health amongst adolescent girls | 327 adolescent girls in grades 7 and 8 from 12 randomly selected schools in Tabriz City  Mean age of control group = 13.1 years  Mean age in first intervention (educating mothers) = 13 years  Mean age in second intervention (education girls) = 13.1 years | Randomized, controlled clinical trial | Attitudes, myths and/or perceptions of menstruation:  Educating girls directly on puberty health improved adolescent girls’ attitudes, however, the study found no positive association between educating mothers and girls’ attitudes.  Experiences of menstruation:  Shame and embarrassment were the most frequent feelings experienced by girls during menstruation. | Moderate |
| (Afsari, Valizadeh et al. 2017) [Iran] | To evaluate the knowledge and practices of adolescent girls about puberty health | 364 adolescent girls in grades 7 and 8 from 5 Districts in Tabriz City  Mean age of girls = 12 ± 3.6 years | Descriptive cross-sectional using a survey | Knowledge of puberty:  The education level of parents showed an association with a girl’s knowledge of puberty.  Sources of information about puberty:  Over two-thirds of girls reported mothers as their main source of information (69%), and the majority (64.5%) said mother was their preferred source of information.  Experience of menstruation:  At the onset of menarche, 22.3% of girls reported fear and worry, 33.1% shame and embarrassment, 14.9% pride / happiness and 6.9% anger. | Weak |
| (Agofure and Iyama 2016) [Nigeria] | To determine the knowledge of puberty, sexually transmitted infections and sexual behaviour among female adolescent students | 120 female adolescent students in junior secondary school between ages 10 and 14 from Agbor, Delta State  100 of these adolescents participated in the quantitative study, 20 in the qualitative study | Descriptive cross-sectional using a survey questionnaire and focus-group discussions (FGD) | Knowledge of puberty:  Most respondents (97.0%) had heard of puberty, with 85.4% able to define it as “the period of maturity”.  Sources of information about puberty:  Girls reported mothers as their primary source of information during puberty. Almost half of respondents in the quantitative study reported school as their primary source of information with one in four respondents reporting parents as their primary source of information. | Weak |
| (Al Omari, Abdel Razeq et al. 2016) [Jordan] | To explore the experience of menarche among Jordanian adolescent girls | 7 girls aged between 11 and 14 years who had attained menarche in the past 6 months, who lived in a 5-km radius of Az-Zarqā | Descriptive cross-sectional using interpretive phenomenological analysis | Sources of information about menstruation:  Participants wrote that information used to prepare them for menarche was “unpleasant” and “frightening”. They reported having no reliable information to prepare them for menarche. Most participants accepted Islamic studies and biology teachers as explainers of the physiological development leading to menstruation.  Experience of menstruation:  Participants did not tell others about menstruation, with one participant using toilet paper instead of sanitary pads to keep her menstruation a secret.  Attitudes, myths and/or perceptions of menstruation:  Participants wrote of the belief that talking about menarche was socially unacceptable and rude | Moderate |
| (Alam, Luby et al. 2017) [Bangladesh] | To examine the association of menstrual hygiene management knowledge, facilities and practice with absence from school during menstruation | 2332 adolescent girls at primary and secondary schools  Mean age = 12.8 years | Descriptive cross-sectional study using a survey | Knowledge of menstruation:  The majority of girls (64%) reported having no knowledge of menstruation before reaching menarche.  Sources of information about menstruation:  The majority of girls who knew of menstruation before menarche heard from a female family member (26%), followed by friends (11%) and teacher (0.64%).  Menstrual hygiene practices:  86% of girls used cloth during menstruation, and 10% used disposable pads. Girls attending urban schools were more likely to use disposable pads. 64% of cloth users washed their cloths with soap; 3% washed their cloths with soap and dried them in the sun.  Experience of menstruation:  41% of girls reported missing school during menstruation, and of those girls, this missing school time represented an average of 16% of the academic year. 59% of these girls reported missing school because they felt uncomfortable sitting beside boys during menstruation, and 31% reported embarrassment. | Weak |
| (Anbumalar and Sasirekha 2016) [India] | To examine menstrual health problems and menstrual management of girls | 209 girls aged 11-14 years from three schools in Chidambaram, Cuddalore District | Descriptive cross-sectional study using a questionnaire | Menstrual hygiene practices:  There was a strong difference in practices between urban and rural girls, with 90% of girls from urban areas using disposable pads and 98% of girls from rural areas using cotton or cloth material.  Attitudes, myths and/or perceptions of menstruation:  Girls had negative attitudes towards menarche: 62.2% of girls in rural areas and 31% of girls in urban areas 31%, arguably due to customs and tradition. No girls reported menarche as desirable. | Weak |
| (Bello, Fatusi et al. 2017) [Nigeria and Kenya] | To assess the cross-cultural and intergenerational reactions of young adolescents and parents to puberty | 66 boys and girls and their families in Ile-Ife (Nigeria) and Nairobi (Kenya) | Descriptive cross-sectional study using qualitative and intergenerational methods | Knowledge of puberty:  Adolescents understood the physical changes during puberty, with more adolescents mentioning breast development as a key change than menstruation. Few male adolescents mentioned the ability for of girls to become pregnant.  Experience of puberty:  Adolescents indicated that they try to hide changes in their bodies from others and sought additional privacy. This included hiding changes from parents.  Source of information on puberty  Parents described trying to meet their children’s needs during puberty in ways their parents had not. They also reported teaching them about morally acceptable behaviour, such as not engaging in pre-marital sex. | Moderate |
| (Bosch, Hutter et al. 2008)  [Bangladesh] | To investigate the perceptions of adolescents and their mothers on markers of reproductive and sexual development | 562 adolescents (307 boys and 255 girls) aged 12 to 16 years in three villages in Matlab  Mean age of girls = 14.1 years  In addition, 18 adolescents (of whom five were married) and five mothers were interviewed in depth | Descriptive cross-sectional using a survey and in-depth interviews | Knowledge of menstruation:  Lack of knowledge about menstruation appeared to be intergenerational, as mothers also lacked information about menstruation. In the in-depth interviews, neither girls nor mothers could tell researchers biological facts about menstruation.  Attitudes, myths and/or perceptions of menstruation:  17% of girls asked questions that were categorized as myths and taboos. Girls reported a range of social and cultural constraints associated with menstruation.  Experience of menstruation:  Adolescent girls were inadequately informed about menarche with 64% of girls reaching menarche in fear. 15% of girls kept their first menstruation a secret.  Sources of information about menstruation  Of those of girls who did talk to someone after their first menstruation, they talked to friend(s) = 19%; sister(s) = 17%; aunt or cousin = 17%; mother = 16%; sister(s)-in-law = 12%; grandmother = 4%. | Weak |
| (Chothe, Khubchandani et al. 2014)  [India] | To document perceptions regarding menstruation and various menstrual restrictions that have been underexplored in India | Students aged 9-13 years in grades 6-8 at three girls’ schools in Pune  318 of 612 possible students submitted questions to the research team | Descriptive cross-sectional study using qualitative methods | Knowledge of menstruation  Students most frequently asked researchers questions about anatomy and physiology (25%).  Attitudes, myths and/or perceptions of menstruation:  Students had substantial doubts about menstruation. Social myths and taboos influenced their menstrual practices.  Experiences of menstruation:  Of the 18% of participants who asked questions about menstrual symptoms, the most commonly questioned symptom was abdominal pain and backache. | Weak |
| (Dabade and Dabade 2017) [India] | To access the knowledge and source of information regarding menstruation among adolescent girls, and to find out practices of menstrual hygiene among them | 230 adolescent girls (123 from the urban field practice area Khaja Bazar and 107 from the rural field practice area Aurad) | Descriptive cross-sectional study | Knowledge of menstruation:  The majority of girls (70.9%) were aware about menstruation before menarche. This was significantly (p=<0.02) higher amongst urban girls (77.2%) than rural girls (63.5%).  Menstrual hygiene practices:  More girls in urban areas (67.5%) than rural areas (52.3%) used sanitary napkins during menstruation. The majority of girls cleaned their external genitalia daily using soap and water.  Attitudes, myths and / or perceptions of menstruation:  67.8% of girls considered menstruation physiological, with 12.6% considering it a curse from God and the remainder responding “don’t know”.  Source of information on menstruation:  The majority of study participants in urban (51.2%) and rural (53.2%) areas got information from their mothers. | Weak |
| (Djalalinia, Tehrani et al. 2012)  [Iran] | To compare different training sources for adolescents’ menstrual health education in Iran | 1,823 female middle school students aged 11-15 years from 15 middle schools in Tehran  Mean age of control group = 11.8 + 1.3 years  Mean age of group trained by school health trainers = 11.65 + 0.51 years | Comparison of control and intervention groups  Outcome assessment 2 years post-intervention | Menstrual hygiene practices:  The trained groups were more likely to take appropriate actions at menarche than controls, but this finding was not significant.  Experiences of menstruation:  Authors reported that negative psychological effects of menarche were lower in the groups that received the intervention. However, insufficient data were reported to calculate an effect size, and findings should be interpreted with caution in light of unclear attrition and analyses.  Sources of information about menstruation:  The trained groups had more sources of information about menstruation than the control group. | Weak |
| (Fazio, Irving et al. 2017)  [Kenya] | To determine whether the availability of menstrual products and ibuprofen would improve examination scores of seventh and eighth grade girls | 99 seventh and eighth grade school girls who were menstruating | A cluster randomized controlled trial with an intervention and control group | Experience of menstruation:  Being provided with sanitary pads and ibuprofen had no significant impact on the association between the experience of menstruation and school performance. | Weak |
| (Haque, Rahman et al. 2014)  [Bangladesh] | The effect of a school-based educational intervention on menstrual health: an intervention study among adolescent girls in Bangladesh | 416 adolescent female students aged 11–16 years who were from three high schools (grades 6–8) and lived with their parents; of those, 52.4% of respondents were aged 11–13 years | Pre-post test | Knowledge of menstruation:  After health education, participants reported a significant improvement in ‘high knowledge and beliefs’ scores compared to baseline.  Menstrual hygiene practices:  Significant improvement was observed in overall good menstrual practices, including improvements in using sanitary pads, frequency of changing pads/cloths per day, drying the used absorbent, methods of disposing of the used absorbent, and cleaning of genitalia.  Experiences of menstruation:  During the follow-up, the participants reported significant improvements in the regularity of their menstrual cycle and fewer complications during menstruation. | Weak |
| (Hennegan, Dolan et al. 2016) [Uganda] | To quantitatively describe girls’ experiences of different menstrual hygiene products. | 205 girls who had attained menarche from eight schools in Kamuli district  Mean age = 14.2 ± 1.12 years | A cross-sectional evaluation | Menstrual hygiene practices:  Girls using reusable pads were more likely to report no problems with changing them at school. They also reported being less disgusted at washing their reusable pads than when using a cloth. | Weak |
| (Hossain, Sharma et al. 2017) [India] | To study adolescent schoolgirls’ knowledge and practices regarding menstrual hygiene | 400 schoolgirls aged 10-19 years in Achrol, Jaipur-Rajasthan | Descriptive observational study | Menstrual hygiene practices:  The majority of girls in the study (85.5%) used sanitary pads, which they changed (73.2%) and disposed of (85.5%). | Weak |
| (Iliyasu, Aliyu et al. 2012)  [Nigeria] | To determine the occurrence, content, and triggers of mother-to-daughter communication about sexual and reproductive health, and to compare menstrual hygiene practices between mothers and their teenage daughters in Ungogo town, a semi-urban community on the out-skirts of Kano city in northern Nigeria | 184 mothers and their unmarried daughters  If more than one teenage daughter was present in the household, the oldest unmarried daughter was interviewed  Daughters’ ages ranged from 11-19 years (mean age = 14.7 + 1.65 years)  Mothers’ ages ranged from 26-54 years (mean age = 38.9 + 4.2 years) | Descriptive cross-sectional using structured interviews and FGDs | Knowledge of menstruation:  69.0% of girls reported discussing menstruation (menstrual hygiene, menses and prayers) with their mothers.  Menstrual hygiene practices:  Daughters more often used sanitary pads during menstruation than mothers (81% vs. 39%); mothers most often re-used a piece of cloth (44%).  Knowledge of puberty:  67.9% of daughters reported discussing breast and body changes with their mother.  Sources of information about menstruation/ puberty:  Most daughters (69%) obtained sexual and reproductive health education from their mothers. Daughters with formal education (13.6%) received this education at school. | Weak |
| (Isguven, Yoruk et al. 2015)  [Turkey] | To determine the level of knowledge and the sources of information about normal puberty and menstrual patterns in schoolgirls from İstanbul, Turkey | 922 randomly chosen schoolgirls  Girls’ ages ranged from 10-17 years (mean age = 14.7 + 2.0 years) | Descriptive cross-sectional using a questionnaire survey | Knowledge of menstruation:  Half of the students did not know the time period between the beginning of puberty and menarche.  Menstrual hygiene practices:  Distribution of daily pad use during a period: <3 pads 10.5%; 3-6 pads 61.9%; >6 pads 12.8%; don't know 14.8%.  Knowledge of puberty:  Girls who had attained menarche were more knowledgeable about puberty, largely through their own experiences.  Sources of information about menstruation/ puberty:  Mothers were the leading source of pubertal information. Students who attained menarche preferred education about puberty to be given by health professionals and to both genders at the same setting. | Weak |
| (Jena, Andalib et al. 2017) [India] | To find out the prevalence of menstrual disorders and health seeking behaviour among adolescent school girls from different socio-economic groups | 597 adolescent schoolgirls aged 10-19 years from Odisha (300 from the international school with higher socio-economic (HSE) backgrounds, 297 from the “tribal residential school” with lower socio-economic (LSE) background)  Mean ages = 13.81 years in the HSE group, 13.62 years in the LSE group | Descriptive cross-sectional using a questionnaire | Knowledge of menstruation:  35% of girls from the HSE group and 89% from the LSE group were not satisfied by their knowledge about menstruation.  Attitudes, myths and / or perceptions of menstruation:  Girls from the HSE group responded that they found menarche disgusting and messy (26.7%) followed by confusing (18.5%) and fearful (17.8%). The LSE group felt fear (25.81%), confusion (18.97%) and sadness (17.41%). Many girls accepted it as normal with the advancement of menstrual years.  Experience of menstruation:  More girls in the HSE group reported associated symptoms (54.67%) than the LSE group (31.97%). This could be due to those in the LSE group being less aware and more accepting of symptoms. | Weak |
| (Jena, Agasti et al. 2017) [India] | To explore the prevalence of menstrual disorders and the sources of knowledge of menstrual problems | 297 adolescent schoolgirls aged 10-19 years from a “tribal residential” school in Odisha  Mean age = 13.62 years | Descriptive cross-sectional using a questionnaire | Knowledge of menstruation:  89% of adolescent girls were not satisfied by their current knowledge and menstruation.  Experiences of menstruation:  One fourth of girls had restrictions on daily activity, and 44.78% were restricted from sports. Of those who were absent during menstruation (9.76%), 52.53% gave the cause as dysmenorrhea.  Sources of information on menstruation:  58.23% said that school education was their main source of information, followed by mother (35.69%). | Weak |
| (Kapadia-Kundu, Storey et al. 2014)  [India] | To describe the results of a school-based, randomized control cluster trial of the gateway moment concept in India, the Saloni pilot intervention, on the nutritional and hygiene practices and health outcomes of girls 11-14 years in Uttar Pradesh, India | 1,200 adolescent girls aged 11-14 years in 30 rural government schools in Hardoi district, rural Uttar Pradesh  At baseline, n = 595; at end-line, n = 601 | Randomized control cluster trial | Menstrual hygiene practices:  The study provided evidence that school-based programs can produce concurrent changes in more than a dozen interrelated health behaviours.  Girls in rural Uttar Pradesh primarily used “cloth pieces” during menstruation rather than sanitary napkins. Girls should change their cloth pieces at least three times a day during menstruation, but constraints such as no toilets at school and lack of privacy at home often prevented them from doing so. Pre-intervention, 29.1% of girls in the intervention and 28.8% of girls in the control groups changed their cloth three times per day.  Post-intervention, 35.1% of girls in the intervention and 18.1% of girls in the control groups changed their cloth three times per day (p<.05). | Moderate |
| (Kheirollahi, Rahimi et al. 2017) [Iran] | To investigate the puberty health status amongst adolescent girls | 152 girls aged 13-15 years from two randomly selected schools in Qom (76 for the intervention, 76 for the control)  Mean age = 13.59 ± 0.87 years | Quasi-experimental intervention | Knowledge of puberty:  Students’ knowledge of puberty, including abnormal signs and hygiene, increased in the intervention group. | Weak |
| (Kumar, Goel et al. 2016) [India] | To assess menstrual problems and treatment-seeking among school going adolescent girls | 655 adolescent girls aged 14-19 years from schools in Chandigarh territory  Mean age = 14.93 ± 1.37 years | Descriptive cross-sectional using a survey | Knowledge of menstruation:  80.0% of girls who had attained menarche had prior knowledge of it.  Sources of information on menstruation:  Mothers were the main source of information (50.8%), followed by teachers (29%).  Experiences of menstruation:  25.3% of girls reported obtaining treatment for menstruation related problems, with shyness reported as the predominant reason for not obtaining treatment.  Attitudes, myths and/or perceptions of menstruation:  The majority of girls (52.8%) considered menstruation to be a normal phenomenon, followed by it being an essential sign of adulthood (31.3%) and essential for fertility in women (29.0%). | Weak |
| (Marván and Alcalá-Herrera 2014)  [Mexico] | To investigate the relationships between menarcheal timing and both menarcheal experience and attitudes toward menstruation in girls in Mexico | 405 post-menarcheal adolescents aged 11-16 years (mean age = 12.98 years) who were attending 3 different public middle schools (first or second grades) in Mexico  City  Participants had to have lived with their biological mothers around the time they experienced menarche, and they had to have reached menarche at least 3 months before they completed the survey | Descriptive cross-sectional using a survey | Knowledge of menstruation:  Early maturers (those who attained menarche before 11 years) were more likely than average maturers (those who attained menarche at 11-12 years) or late maturers (those who attained menarche at 13+ years) to state they had not known what they should do at the moment they got their first period (p<.01), that they had not felt prepared to start menstruating (p<.05), and that they thought they must keep secret the fact of already having had their first period (p<.05).  Attitudes, myths and/or perceptions of menstruation:  Attitudes measured by the Mexican post-menarcheal form of the Adolescent Menstrual Attitude Questionnaire (AMAQ). Early maturers scored higher than both average and late maturers on Secrecy and Negative attitudes. Late maturers showed more positive attitudes toward menstruation than their peers (p<.01).  Experiences of menstruation:  Early maturers were also the most likely to have felt scared (p<.05), worried (p<.05) and sad (p<.05). Late maturers showed more positive attitudes toward menstruation than their peers (p<.01). | Weak |
| (Marván and Molina-Abolnik 2012)  [Mexico] | To investigate the relationships between menarcheal timing and (1) menarcheal experience and (2) attitudes toward menstruation in girls in Mexico | 602 female students in 10 different public schools in Mexico City who were attending grades equivalent to grades 5-10 in North American schools  145 participants (24.1%) were aged 11-12 years  248 participants (41.2%) were aged 13-14 years  209 participants (34.7%) were aged 15-16 years | Descriptive cross-sectional using a survey | Knowledge of menstruation:  94% had discussed menstruation with their mother prior to first period, of whom most had discussed hygiene and body function (89% and 82% respectively). Less than half had discussed emotional aspects or physical sensations (39% and 45% respectively).  Attitudes, myths and/or perceptions of menstruation:  Regarding menstrual attitudes, adolescents scored highest on negative feelings and secrecy rather than on positive feelings.  Experiences of menstruation:  Most participants knew what they should do when they experienced their menarche, though only 39% stated they had felt prepared to start menstruating. Participants who had previously discussed the emotional aspects of menses with their mothers were more likely to claim they had felt prepared to start menstruating when they got their first period (OR = 3.45). The fact that adolescents felt prepared to start menstruating predicted positive attitudes toward menstruation. | Weak |
| (Mason, Nyothach et al. 2013)  [Kenya] | To examine girls’ attitudes, experiences, and concerns around menarche and  menstruation as part of a baseline evaluation for a randomised controlled pilot study examining the potential benefits of different menstrual solutions for adolescent primary schoolgirls in rural western Kenya | 120 schoolgirls aged 14–16 years (mean age = 14.3 years) who attended schools eligible for the Menstrual Solutions Study (the bigger project under which this study was nested) | Descriptive cross-sectional using FGDs | Knowledge of menstruation:  Girls were unprepared for menstruation and demonstrated poor knowledge.  Menstrual hygiene practices:  Sanitary pads were valued, but resource and time constraints result in prolonged use causing chafing. Alternatives included rags and grass. Girls reported that ‘other girls’ participated in transactional sex to buy pads and received pads from boyfriends.  Attitudes, myths and/or perceptions of menstruation:  Menarche was widely perceived as a sign of being grown up or mature, between child and adulthood, and a marker of sexual attraction to boys, with implications for marriage. Narratives repeatedly mentioned the importance of secrecy around menstruation. Fear was the prevailing emotion associated with menstruation and a major reason for the need for secrecy, linked to shame surrounding leaking blood or keeping clean.  Experiences of menstruation:  Girls devised practical methods to cope with menstrual difficulties, often alone since parental and school support of menstrual needs was limited. Girls admitted ‘other girls’ were absent from school during menstruation, due to physical symptoms or inadequate sanitary protection.  Sources of information about menstruation:  Information was usually provided to girls by other females (mothers, older sisters, fellow pupils). Some girls had been taught in science class. | Strong |
| (Mishra, Dasgupta et al. 2017)  [India] | To understand the relationship of socioeconomic characteristics, menstrual hygiene practices and gynaecological problems among adolescent girls residing in rural and urban areas in West Bengal | 715 girls aged between 10-19 who had attained menarche at least two years before the survey.  Mean age = 14.19 ± 1.51 years | Descriptive cross-sectional using survey | Knowledge of menstruation:  Urban participants reported higher levels of information from mothers pertaining to menstruation prior to menarch (83.25%) than rural participants (59.49%).  Menstrual hygiene practices:  There were significant differences between urban and rural participants. Urban participants cleaned their genitals and used pads at higher frequencies than rural participants (no participants in either group used tampons or menstrual cups).  Experiences of menstruation:  Over 90% of urban and rural participants reported experiencing some kind of restriction imposed on them during menstruation, mainly from parents. | Weak |
| (Montgomery, Hennegan et al. 2016) [Uganda] | To assess the impact of providing reusable sanitary pads and puberty education on school attendance | 1008 school age adolescents in grades 3-5 in Kamuli district. Intervention groups were provision of puberty education; provision of reusable sanitary pads; provision of puberty education and reusable sanitary pad; and control.  Mean age = 11.44 years | Cluster quasi-randomized controlled trial | Knowledge of menstruation:  Authors found no difference in girls’ knowledge of menstruation at follow-up. They noted the measurement of this knowledge was poor.  Attitudes, myths and / or perceptions of menstruation:  There was no significant difference in psychosocial outcomes across interventions at follow-up. Only a small number of girls completed follow-up survey. | Weak |
| (Moodi, Zamanipour et al. 2013)  [Iran] | To evaluate the effect of educational program for puberty health on improving intermediate and high school female students' knowledge in Birjand, Iran | 302 female intermediate (n = 151) and high school (n = 151) students were selected through randomized cluster sampling  Mean age = 12.9 ± 1.1 years | Quasi-experimental study using a questionnaire | Knowledge about puberty:  The mean [undefined] knowledge level among intermediate students was 5.03 ± 3.7 before intervention and 10.8 ± 4.8 after intervention. Among high school students, the mean [undefined] knowledge scores were 4.1 ± 2.3 pre-intervention and 8.7 ± 3.8 post-intervention. Both groups experienced a significant difference between pre- and post-intervention results (p<0.001). | Weak |
| (Mumtaz and Ansari 2016) [Pakistan] | To assess the beliefs and practices relating to menstrual hygiene | 416 adolescent schoolgirls from Bharakahu Islamabad | Descriptive cross-sectional using survey | Menstrual hygiene practices:  49.8% of participants used old cloths during menstruation, as opposed to sanitary pads (16.8%). The authors found 56.8% hygiene practices to be graded as very poor.  Experiences of menstruation:  The majority (92.3%) of girls did not attend school during menstruation, and 77.4% were restricted from religious activities. Most girls (90.8%) responded that they were not likely to consult someone over menstrual related complications. | Moderate |
| (Nagaraj and Konapur 2016) [India] | To assess the effect of health education on knowledge, attitude and practices regarding menstruation | 304 adolescent schoolgirls within the rural field practice area of Kempegowda Institute of Medical Sciences, Bangalore  Mean age = 14.27 ± 0.98 years | Pre-post test using a questionnaire | Knowledge of menstruation:  Only a small proportion (29.93%) of girls were aware of menstruation before menarche.  Sources of information on menstruation:  Mothers were the primary source of information (47.03%), followed by sisters (25.65%), friends (15.46%), and teachers (8.55%).  Menstrual hygiene practices:  A small proportion of girls (26.3%) in the study used sanitary pads. | Weak |
| (Phillips-Howard, Nyothach et al. 2016) [Kenya] | To study the effect of menstrual hygiene on girls’ school and health outcomes | 751 adolescent schoolgirls from the Gem District in Siaya County (200 in the control group, 256 given pads, 188 given cups)  Mean age = 14.6 ± 0.7 years | Open cluster randomized controlled pilot study | Menstrual hygiene practices:  Reproductive tract infections were prevalent across controls (between 20-30%). Prevalence differences were greatest among girls who had received the intervention longer. | Moderate |
| (Pratibha, Nawaz et al. 2016) [India] | To explore the practices related to menstrual hygiene, and the impact and challenges of these. | 346 adolescent schoolgirls from two schools in the practice area of a medical college in Central Karnataka  Mean age = 13.93 ± 0.811 years | Descriptive cross-sectional using a survey | Menstrual hygiene practices:  The majority of girls (53.8%) used sanitary napkins, with 46.2% using cloth. Just over a third of girls (37.6%) reported regular changing of their absorbent.  Knowledge of menstruation:  44.2% of girls reported not knowing the recommended method of washing and drying menstrual absorbents.  Experience of menstruation:  The majority of girls (68.8%) said they did not miss school for fear of punishment, whilst 31.2% remained absent due to lack of adequate facilities to manage menstrual hygiene. | Weak |
| (Ramachandra, Gilyaru et al. 2016) [India] | To explore knowledge and practices regarding menstrual hygiene | 550 adolescents from schools in Bangalore city  Mean age = 13.98 years | Descriptive cross-sectional using a survey | Knowledge of menstruation:  A third (33.27%) of adolescents in urban environments had awareness of menstruation prior to menarche.  Sources of information on menstruation:  Teachers were not considered a good or preferred source of information. More commonly reported sources were mothers, followed by friends and sisters. | Weak |
| (Saghi, Mirghafourvand et al. 2016) [Iran] | To determine adolescent’s knowledge and attitude towards puberty health | 1017 adolescents from Tabriz City  Mean age = 13.1 ± 0.7 years | Descriptive cross-sectional study using a survey | Knowledge of puberty:  The majority of adolescents (85.8%) had moderate knowledge of puberty, with the question most frequently answered correctly (91.2%) related to eating behaviours. The question least frequently answered correctly (9.1%) involved normal menstrual cycle intervals.  Attitudes, myths and / or perceptions of menstruation:  Most (90%) of participants had positive attitudes towards menstruation. Attitudes were more positive among those in better economic situations. | Weak |
| (Shah, Nair et al. 2013)  [India] | To study adolescent girls’ knowledge regarding menstruation and menstrual practices; their quality of life; experience and satisfaction with three different kinds of menstrual pads (old cloths, a new soft cloth (*falalin*), and subsidized sanitary pads); and any differences in symptoms of reproductive tract infection using these three kinds of pads | 164 unmarried post-menarchal adolescent girls (school-going and non-school-going) living in an under-privileged area in all eight project villages in India  Mean age = 13.7 years | Pre-post design | Knowledge of menstruation:  65/164 girls reported having knowledge about menstruation before first menses. Knowledge of source of menstrual blood: lower abdomen = 14/164, urinary site = 66/164, uterus = 20/164, lower side = 14/164, don't know = 50/164. Path of urine and menstruation: same = 81/164, separate = 62/164, don't know = 21/164.  Menstrual hygiene practices:  Both *falalin* cloths and sanitary pads were quickly shown to be acceptable to a large proportion of girls. *Falalin* cloth was preferable to sanitary pads because it is easily washable, reusable, soft, and has good absorbing capacity. Most importantly, *falalin* cloth is easily available at the community level at low cost and is environmentally friendly as it is disposed of easily by burning.  Experiences of menstruation:  The use of *falalin* cloths and sanitary pads resulted in clear improvements in quality of life issues related to menstruation.  Sources of information about menstruation:  Mothers (29/65) were the main source of information about menstruation, followed by friends (18/65). | Weak |
| (Sharma, Choudhary et al. 2016) [India] | To assess the knowledge and practices of menstrual hygiene, and the restrictions faced during menstruation | 150 adolescent girls attending secondary schools near the primary health centre, Agroha, Hisar | Descriptive cross-sectional study | Knowledge of menstruation:  Only 4.7% of girls did not know about menstruation before menarche.  Sources of information on menstruation:  Most girls obtained information on menstruation from their mothers (52.5%), followed by friends (24.5%) and sisters (23.1%).  Experience of menstruation:  The majority (92%) of girls were restricted from religious matters during menstruation. 45.3% of girls were restricted from cooking and handling food items, and 22.7% were restricted from outdoor activities. | Weak |
| (Sharma and Moktan 2017) [Nepal] | To identify menstrual hygiene knowledge and practice | 56 adolescent school girls from one secondary school in Siraha district | Descriptive cross-sectional study | Knowledge of menstruation:  Most girls (83.9%) had adequate knowledge of menstrual hygiene. Mothers were the main source of knowledge.  Menstrual hygiene practices:  Less than half (45.5%) of girls had an appropriate level of menstrual hygiene practices.  Experience of menstruation:  One fifth (21%) of respondents were at least partly restricted from social functions during menstruation. | Weak |
| (Sharma, Negi et al. 2015)  [India] | To assess knowledge and practice regarding menstrual hygiene before and after teaching program among adolescent girls in India | 50 adolescent secondary school girls (n = 25 intervention; n = 25 control) studying in selected high school of Bhaniyawala Post,  Dehradun District  Mean age of participants = 13.88 + 1.5 years | "A true experimental study" that used a pre- and post-test questionnaire | Knowledge of menstruation:  Researchers noted that the level of knowledge and practice regarding menstrual hygiene of subjects who participated in the educational program was significantly better than that of the control group.  Menstrual hygiene practice:  Frequency of changing sanitary pads, reusing cloth after washing, and reported cleanliness of genitalia during menstruation were significantly (p=0.01) higher among education programme participants.  Experiences of menstruation:  Mean age of menarche 12.74 ± 0.98 years. Menstrual period duration: 3-5 days (60%); 5-7 days (22%), >7 days (18%). 36% had dysmenorrhoea, 26% presented with breast tenderness, 6% had both (dysmenorrhoea & breast tenderness), and 32% had no complaints.  Sources of information about menstruation:  The majority (78%) of participants received information from their mothers; 14% from female friends and 8% from teachers. | Weak |
| (Shoor 2017) [India] | To study the knowledge and attitude towards menstruation, menstrual hygiene practices, menstrual morbidity and health seeking behaviour | 452 school girls whose parents had given consent from the urban field practice area of Sri Siddhartha Medical College  Mean age = 13.05 ± 0.09472 years | Descriptive cross-sectional | Knowledge of menstruation:  The majority of girls (62.61%) did not know about menstruation before menarche. 62.39% of participants knew menstruation only occurs in females.  Menstrual hygiene practices:  Most girls used sanitary pads during menstruation (84.12%), and the majority changed their pads 2-3 times daily.  Attitudes, myths and /or perceptions of menstruation:  Just under half (49.36%) of girls reported celebrations upon attaining menarche, with a third (35.32%) reporting being scared.  Experiences of menstruation:  Few (11.08%) girls reported having no restrictions during menstruation. Of those who had restrictions, the majority of restrictions involved religious practices (53.98%) and were imposed by family members (54.51%). | Weak |
| (Sridhar and Gauthami 2017) [India] | To observe the menstrual health of adolescent girls, considering cultural factors | 425 adolescent school girls aged 10-19 years, including married, antenatal and postnatal, from 18 habitations of Achampet Mandal | Descriptive cross-sectional study | Menstrual hygiene practices:  Of girls who were illiterate, the majority (55.1%) had non-sanitary practices. This was also true for girls whose mothers were illiterate (52.2%).  Experiences of menstruation:  The main restriction experienced by girls was religious (86.81%), with 20.2% of girls reporting food restrictions and 21.6% of girls reporting restrictions from routine household activities. One fifth (20%) reported school absenteeism. | Weak |
| (Su and Lindell 2016) [China] | To study the effects of a nursing intervention on the menstrual health of girls | 116 girls in Wuhan (60 girls in the intervention group from School A, 56 girls in the control group from School B)  Mean age = 12.26 ± 0.56 years | Quasi-experimental pre-post test | Knowledge of menstruation:  The authors found a significant difference in girl’s knowledge of menstruation post-intervention compared with the control group.  Attitudes, myths and / or perceptions of menstruation:  There was a statistically significant decrease in the attitude that menstruation was debilitating and bothersome in the intervention group. | Weak |
| (Um, Yusuf et al. 2010)  [Nigeria] | To examine the knowledge and practices of adolescent school girls in Kano, Nigeria around menstruation and menstrual hygiene | 400 adolescent female secondary school students aged 10-19 years (mean age = 14.4 + 1.2 years) | Descriptive cross-sectional using a survey | Knowledge of menstruation:  Most participants (87.5%) were graded as having fair knowledge of menstruation, although they were deficient in specific knowledge areas. Only 4.0% of participants received a "good" grade on knowledge of menstruation.  Menstrual hygiene practices:  The majority (88.7%) of students identified having good practice of menstrual hygiene. Most of them used sanitary pads as an absorbent during their last menses (93.8%) and increased the frequency of bathing (72.5%). Students reported changing their menstrual dressings from 1-5 times per day (mean = 2.6 + 0.8).  Experiences of menstruation:  The mean age at menarche was 12.9 ± 0.8 years.  Sources of information about menstruation:  First source of information on menstruation and menstrual hygiene was parents (35.3%); 14.3% learnt about menstruation at school, and the remainder from friends (20.0%) or sisters (21.5%). | Weak |
| (Valizadeh, Assdollahi et al. 2017) [Iran] | To compare the effects of education of mothers and their daughters on puberty hygiene knowledge and practices | From high schools sampled from Tabriz city (124 girls in the first intervention focused on girl’s education, 120 girls in the second focused on mothers’ education, 120 girls in the control)  Mean age = 12 ± 3.6 years | Randomized controlled clinical trial. | Knowledge of puberty:  There was no significant difference in scores on knowledge prior to intervention. Post-intervention, the mean score on knowledge was significantly higher in both intervention groups compared to the control group. | Strong |

2. Summary table of studies that included an intervention, including description of the intervention, evaluation, intervention claims, and reported limitations (n=15)

| **Author, year** | **Intervention** | **Evaluation** | **Intervention claims** | **Reported limitations** |
| --- | --- | --- | --- | --- |
| (Afsari, Mirghafourvand et al. 2017) | Software: There were two intervention groups aimed at educating mothers and girls respectively, and one non-intervention group.  For the mother’s education group, half-hour lectures were given on puberty hygiene using booklets. Each mother was given an educational booklet and two pamphlets. In the girl’s education group, education packages were also provided.  The intervention was performed over two months. | Participants were asked to fill out attitude questionnaires before the intervention and then again two months after the intervention. | Educating girls directly improved their attitude scores at a statistically significant level. Educating mothers had no significant impact on the attitudes of students. | The short duration of the educational sessions was reported as a limitation, including insufficient frequency and lack of follow up of questions after the sessions had been completed. |
| (Djalalinia, Tehrani et al. 2012) | Software: Menstrual health education (of which the content, duration and format are unclear)  Students were allocated randomly to three groups: trained by parents, trained by schools’ health trainers, and control (no training). | Researchers first conducted a survey on the menstrual health of participants. Participants were then randomized into three groups.  Following a two-year training program, adolescents’ knowledge of puberty and menstrual health were assessed. | The authors reported positive claims for the effects of the intervention on the outcomes.  Menstrual health training from parents and teachers led to better experiences of menarche and higher use of sanitary pads or cotton; however, based on this experience, the authors concluded that adolescents’ health promotion is best addressed in school-based interventional programmes. | Missing data values, particularly for questions that rely upon experience of menarche  The 9-month school calendar used in this intervention is less than the 12-month minimum recommended by the mentoring field. |
| (Fazio, Irving et al. 2017) | Software: Puberty education for both the intervention and control groups  Hardware: The intervention group was given sanitary pads and ibuprofen.  Students from fourteen schools were randomly allocated into two groups: intervention (n=45) and control (n=54). Both groups received puberty education, whilst the intervention group also received sanitary pads and ibuprofen.  No details of how the intervention was administered were presented. | Standard school examinations were used to measure the success of the intervention on performance. School examination results from the first school term were taken as a baseline and compared to results in the second and third school terms. | The difference between test scores from the intervention and control groups were not significant across the terms; thus, the intervention was assumed to have had no impact. | Limited by potential cultural and socioeconomic factors, as well as unidentifiable confounders |
| (Haque, Rahman et al. 2014) | Software: Trained research assistants (RAs) delivered a 6-month menstrual hygiene educational intervention among schoolgirls.  The training was delivered using a field manual in the Bangla language.  Menstrual education focused on menstrual hygiene knowledge, beliefs and behaviours, menstrual disorders, and restrictions on menstruating adolescents. Educational materials were developed by an obstetrician/gynaecologist and were culturally acceptable to the girls. Girls received twelve 45-minute lessons once every 15 days. The RAs also used clean cloths and pads for demonstrations. 12 FGDs were conducted in the schools so that RAs and adolescent girls could become well acquainted with each other. | FGDs were conducted to qualitatively evaluate the effectiveness of the intervention. After 6 months of intervention, follow-up data were collected in the schools using the same questionnaire as at baseline on menstrual hygiene knowledge, beliefs and practices, types of complications, and restrictions on menstruating adolescents. RAs visited the homes of any students who were not available at school during the follow-up data collection. | The authors made positive claims for the effects of the intervention on the outcomes.  A school-based health education programme produced significant changes in girls’ knowledge of menstruation, beliefs and practices of menstrual hygiene; however, the intervention did not lead to significant improvements in menstrual restrictions (e.g. visiting holy sites, doing household work), indicating the widespread nature of menstrual taboos and socio-cultural beliefs. | Data were based on self-reported outcomes that may differ from actual behaviour.  Obtaining data on menstrual complications from participants rather than medical records might have led to bias.  Dysmenorrhea was not assessed with a pain scale but rather with the report of any menstrual pain in the abdomen, groin, and lumbar regions.  Despite attempts to standardise the intervention, environmental factors (e.g. abilities of RAs) may have affected the outcomes. |
| (Hennegan, Dolan et al. 2016) | Software and Hardware: Four intervention groups were established: puberty education alone; reusable pad provision alone; puberty education and reusable pad provision; no intervention/control.  The education involved a 1.25-hour session following the Straight Talk guide. AFRIpads were used for the groups who received sanitary pads. | Data for evaluation were taken from the final, follow-up survey of the ‘Menstruation and The Cycle of Poverty’ controlled trial. | Reusable pads were considered more reliable and manageable than the existing absorbents used by girls.  There was no association between reusable pads and puberty education and an improvement in school attendance. | Using local RAs to administer the surveys left the responses vulnerable to social-desirability effects.  The sample size was insufficient to assess outcomes across all absorbents. |
| (Kapadia-Kundu, Storey et al. 2014) | Software: The Saloni pilot intervention, implemented for one year, was designed as an addition to the existing government school health programme for adolescent girls (Saloni Swasth Kishori Yojna; SSKY). A prevention model that includes Sadharanikaran, an ancient Indian theory of communication, guided intervention development, including the design of the teacher's manual and the Saloni diary. The 10 one-hour sessions were conducted monthly and promoted 19 behaviours: 5 health seeking behaviours, 6 nutrition behaviours, 3 reproductive health (RH) behaviours and 5 hygiene behaviours. Teachers were trained to encourage girls to share what they learned with their parents after every session. Role-plays were used to demonstrate how to initiate Saloni discussions with parents. The intervention also used diaries to engage adolescent girls, articulate new social and behavioural norms and reinforce daily practice of protective nutrition and hygiene behaviours.  Intervention group = SSKY + pilot Saloni curriculum  Control group = SSKY only | A two-level, nested RCT with the unit of randomization being the block with 15 schools in the intervention arm and 15 schools in the control arm | The authors made positive claims for the effects of the intervention on the outcomes.  School-based education programmes can produce change across multiple health issues. | The study’s short timeframe did not permit measurement of long-term impact.  Researchers could not measure the extent to which participants sustained behaviours beyond the intervention period. |
| (Kheirollahi, Rahimi et al. 2017) | Software: There were three topics covered in 45-minute sessions that consisted of lectures, questions, and answers.  Topics addressed the importance of puberty health and nutrition during puberty, the negative consequences of the lack of puberty health, and the benefits related to good puberty behaviour. | Before the intervention, students in the intervention and control groups completed a questionnaire that they then undertook again two months after the end of the sessions. | The authors found that the mean score of knowledge was higher in the intervention group post-intervention than in the control group.  There was no statistically significant difference in the post-test on perceived susceptibility, severity, benefits, and barriers of puberty health. | The authors noted limitations, including that: the questionnaires were completed via self-reports, students did not always pay attention during education sessions, and school principle cooperation during the intervention was limited. |
| (Montgomery, Hennegan et al. 2016) | Hardware and software: Girls receiving pads were provided with a pack of AFRIpads, 3 pairs of underwear, and a sachet of soap. Girls were also taught about correct use and cleaning by local non-governmental organization assistants.  Puberty education was provided by locally trained community health nurses. The education lasted 1.25 hours and followed the Straight Talk training guide, covering puberty changes, menstruation, pregnancy, life skills, prevention of HIV, strategies for avoiding sexual assault, healthy relationships, and friendship formation. | Baseline attendance was taken prior to the randomization, with follow up attendance taken post intervention.  Surveys were administered at baseline, mid-point, and the conclusion of the study. These surveys covered menstrual practices, psychosocial outcomes, and wellbeing. | Both education and giving out reusable sanitary pads were equally as effective in improving school attendance compared to the control group. | School attendance was a major barrier to the intervention with girls dropping out. This could have been both in real terms, but also in the change of measuring attendance during the intervention leading to potential bias. Educational sessions were run only once, so girls absent on the day missed the intervention.  The intervention delivery was also reported as weak, with limitations on the reliability of the results found. Pads were found to have not been delivered in a consistent way, with delivery of the second batch of pads delayed. |
| (Moodi, Zamanipour et al. 2013) | Software: Trained instructors taught all students identical educational content. The time of training in each class was delivered in two 45-minute sessions. Students also received educational pamphlets about the required subject. | Researchers evaluated the intervention using a pre-test and a post-test. Data collection used a questionnaire based on course objectives and included 20 one-score multi-choice questions for assessment of knowledge.  A month after the intervention in each school, the questionnaires were distributed once again among the students and then collected for analysis. | The authors made positive claims for the effects of the intervention on the outcomes.  The intervention resulted in increased mean scores of knowledge about puberty. | Unreported |
| (Nagaraj and Konapur 2016) | Software: Health educational sessions were conducted, with 20 students in each session. These sessions included audio-visual aids and contained material on menstruation, common health problems, and hygiene practices. Sessions were followed by clarification of any student doubts. | A multiple-choice questionnaire was used to test knowledge, attitude, and practices before and after the intervention. | Health education significantly improved knowledge, attitude, and practices regarding menstruation and hygiene.  There was an improvement in hygiene practices post-health education, for example, in washing and drying menstrual cloths. | Out-of-school girls could not be involved in the study. Researchers found that very few mothers participated during the health education sessions. |
| (Phillips-Howard, Nyothach et al. 2016) | Hardware and software: Each intervention group received puberty and hygiene training. The groups with cups (Mooncups) and pads received product-specific training. Girls in the cup intervention were each provided one menstrual cup with instructions on insertion and cleaning. | Kenyan registered nurses were used to follow up with the girls on a weekly basis, documenting menstruation, intervention use, etc. Girls absent for a term were visited at home to ascertain the reason.  At the end of the study, vaginal swabs were taken by girls to check for reproductive tract infections and sexually transmitted infections. | Providing menstrual cups and sanitary pads for one school year was not associated with a significant impact on school dropout rates. | The limited research on using menstrual cups and the impact of poor school hygiene meant that schools were selected based on the pupil-latrine ratio, with separate toilets for girls. This limited the generalizability of the study. |
| (Shah, Nair et al. 2013) | Hardware: Researchers introduced *falalin* cloths, followed by sanitary napkins, among girls who were using old cloth at the baseline. New *falalin* cloths (reused for three cycles) were offered to all girls for three months at a subsidized price through village-based accredited social health activists (ASHAs). Subsequently, sanitary pads were offered for another three months at subsidized price, also via ASHAs.  Programme supervisors visited the study villages monthly to ensure the intervention was being implemented as planned. | Survey data were collected at baseline and again three months after introducing each of the two kinds of absorbent pads. | The researchers made positive claims for the effects of the intervention on the outcomes.  Whilst *falalin* cloths and sanitary pads were both acceptable to adolescent girls, *falalin* cloths were preferable. Access to sanitary pads and *falalin* cloths should be scaled up and promoted in rural India.  *Falalin* cloths provided an opportunity to improve menstrual hygiene, though additional research is required to assess effectiveness. | Unreported |
| (Sharma, Negi et al. 2015) | Software: The experimental group underwent an educational training programme on menstrual hygiene. The structured educational content was prepared after a review and with the help of experts from nursing and medical professions. | Researchers administered a questionnaire on day one to collect pre-test data from both groups. They collected post-test from both groups 15 days after the intervention ended. | The researchers made positive claims for the effects of the intervention on the outcomes.  The menstrual hygiene educational programme effectively enhanced menstrual health and produced better knowledge and practice regarding menstrual hygiene. | Unreported |
| (Su and Lindell 2016) | Software: Educational sessions were conducted at The intervention school, divided into four groups, received educational sessions lasting 45-minutes long five times a week. Teaching was interactive and included lectures, group discussions, visual aids, and materials. Educational sessions were aimed at daily life concerns. | Researchers conducted pre- and post-tests to measure the effectiveness of the intervention. Following the post-test, girls in the control were offered the same educational sessions as the intervention group. | The study found that the intervention improved girls’ menstrual health and improved their confidence during menstruation and attitudes towards menstruation. It also increased pain relief practice. | Using self-reporting data meant the authors needed to assume that the answers reflected actual behaviour.  As the study did not use randomization, its generalizability is limited. |
| (Valizadeh, Assdollahi et al. 2017) | Software: The schools allocated to educating mothers sent invitations via their daughters to attend a class. The 30-minute class used material in a booklet that the mother was then given along with two educational pamphlets.  Students received an education package that included the booklet “How do I reach puberty”. The booklet included questions and answers on menstrual hygiene, nutrition, sports, and psyche.  No intervention took place in the control group. | All girls (intervention and control) completed a questionnaire containing questions on knowledge and practice two months after the interventions had taken place. | The intervention did not show any positive effects on educating girls or their puberty hygiene practices.  The intervention suggested mothers should participate in teaching puberty hygiene to their daughters. | The short duration of the intervention was limiting;therefore, the authors recommend a longer study duration.  The effect of different age groups among students was not tested.  The lack of education sessions following the intervention and the insufficient frequency were considered to be limiting factors. |

**References**

Adhikari, P., B. Kadel, S. I. Dhungel and A. Mandal (2007). "Knowledge and practice regarding menstrual hygiene in rural adolescent girls of Nepal." Kathmandu Univ Med J (KUMJ) **5**(3): 382-386.

Afsari, A., M. Mirghafourvand, S. Valizadeh, M. Abbasnezhadeh, M. Galshi and S. Fatahi (2017). "The effects of educating mothers and girls on the girls' attitudes toward puberty health: a randomized controlled trial." Int J Adolesc Med Health **29**(2).

Afsari, A., S. Valizadeh, S. Fatahi, M. Abbasnezhad and M. Assdollahi. (2017). "Predictors of Knowledge and Practice of Girl Students about Puberty Health." International Journal of Pediatrics **5**(7): 5229-5236.

Agofure, O. and M. Iyama (2016). "Knowledge of puberty, sexually transmitted infections, and sexual behavior among very young female adolescent students' 10-14 years in Agbor Metropolis, Nigeria." Nigerian Journal of Health Sciences **16**(1): 27.

Al Omari, O., N. M. Abdel Razeq and M. M. Fooladi (2016). "Experience of Menarche Among Jordanian Adolescent Girls: An Interpretive Phenomenological Analysis." J Pediatr Adolesc Gynecol **29**(3): 246-251.

Alam, M.-U., S. P. Luby, A. K. Halder, K. Islam, A. Opel, A. K. Shoab, P. K. Ghosh, M. Rahman, T. Mahon and L. Unicomb (2017). "Menstrual hygiene management among Bangladeshi adolescent schoolgirls and risk factors affecting school absence: results from a cross-sectional survey." BMJ Open **7**(7): e015508.

Anbumalar, A. and B. Sasirekha (2016). "Menstrual Health Problems and Menstrual Management of Adolescent Girls." International Journal of Nursing Education and Research **4**(4): 411-412.

Bello, B. M., A. O. Fatusi, O. E. Adepoju, B. W. Maina, C. W. Kabiru, M. Sommer and K. Mmari (2017). "Adolescent and parental reactions to puberty in Nigeria and Kenya: A cross-cultural and intergenerational comparison." Journal of Adolescent Health **61**(4): S35-S41.

Bosch, A. M., I. Hutter and J. K. van Ginneken (2008). "Perceptions of adolescents and their mothers on reproductive and sexual development in Matlab, Bangladesh." Int J Adolesc Med Health **20**(3): 329-342.

Chothe, V., J. Khubchandani, D. Seabert, M. Asalkar, S. Rakshe, A. Firke, I. Midha and R. Simmons (2014). "Students' perceptions and doubts about menstruation in developing countries: a case study from India." Health Promot Pract **15**(3): 319-326.

Dabade, K. J. and S. K. Dabade (2017). "Comparative study of awareness and practices regarding menstrual hygiene among adolescent girls residing in urban and rural area." International Journal Of Community Medicine And Public Health **4**(4): 1284-1288.

Djalalinia, S., F. R. Tehrani, H. M. Afzali, F. Hejazi and N. Peykari (2012). "Parents or School Health Trainers, which of them is Appropriate for Menstrual Health Education?" International Journal of Preventive Medicine **3**(9): 622-627.

Fazio, J.C., M. Irving, F. Marquez, M. Deissinger, A. Tomedi and C. Schmitt (2017). "The Effect of Sanitary Pads and Menstrual Symptom Management on School Performance of Adolescent Girls in Rural Kenya: A Cluster Randomized Trial." Annals of Global Health 83(1): 100.

Haque, S. E., M. Rahman, K. Itsuko, M. Mutahara and K. Sakisaka (2014). "The effect of a school-based educational intervention on menstrual health: an intervention study among adolescent girls in Bangladesh." BMJ Open **4**(7): e004607.

Hennegan, J., C. Dolan, M. Wu, L. Scott and P. Montgomery (2016). "Schoolgirls' experience and appraisal of menstrual absorbents in rural Uganda: a cross-sectional evaluation of reusable sanitary pads." Reprod Health **13**(1): 143.

Hossain, S., P. Sharma and V. Sen (2017). "A Study of the Knowledge and Practice Regarding Menstrual Hygiene in Rural Adolescent School Going Girls in an Indian Cosmopolitan City." Journal of Pharmacy Practice and Community Medicine **3**(3): 185-187.

Iliyasu, Z., M. H. Aliyu, I. S. Abubakar and H. S. Galadanci (2012). "Sexual and reproductive health communication between mothers and their adolescent daughters in northern Nigeria." Health Care Women Int **33**(2): 138-152.

Isguven, P., G. Yoruk and F. M. Cizmecioglu (2015). "Educational Needs of Adolescents Regarding Normal Puberty and Menstrual Patterns." Journal of Clinical Research in Pediatric Endocrinology **7**(4): 312-322.

Jena, P., N. Agasti, S. Andalib and K. Khandelwal (2017). "Menstrual problems and health awareness of tribal adolescent schoolgirls of Odisha-A cross-sectional study." Journal of Evolution of Medical and Dental Sciences **6**(51): 3917-3921.

Jena, P., S. Andalib, S. Khuntia and A. Mishra (2017). "Spectrum of menstrual disorder and health conciousness of adolescent school going girls: A comparative study between the extremes of two socio-economic group." Indian Journal of Obstetrics and Gynecology Research **4**(3): 235-239.

Kapadia-Kundu, N., D. Storey, B. Safi, G. Trivedi, R. Tupe and G. Narayana (2014). "Seeds of prevention: the impact on health behaviors of young adolescent girls in Uttar Pradesh, India, a cluster randomized control trial." Soc Sci Med **120**: 169-179.

Kheirollahi, F., Z. Rahimi, S. Arsang-Jang, G. Sharifirad, P. Sarraf and Z. Gharlipour (2017). "Puberty Health Status among Adolescent Girls: A Model-based Educational Program." International Journal of Pediatrics-Mashhad **5**(7): 5369-5378.

Kumar, D., N. K. Goel, M. K. Sharma and G. Kaur (2016). "Menstrual problems of school going unmarried adolescent girls and their treatment seeking behavior in Chandigarh, India." International Journal Of Community Medicine And Public Health **3**(11): 3106-3116.

Marván, M. L. and V. Alcalá-Herrera (2014). "Age at Menarche, Reactions to Menarche and Attitudes towards Menstruation among Mexican Adolescent Girls." Journal of Pediatric and Adolescent Gynecology **27**(2): 61-66.

Marván, M. L. and M. Molina-Abolnik (2012). "Mexican adolescents' experience of menarche and attitudes toward menstruation: role of communication between mothers and daughters." J Pediatr Adolesc Gynecol **25**(6): 358-363.

Mason, L., E. Nyothach, K. Alexander, F. O. Odhiambo, A. Eleveld, J. Vulule, R. Rheingans, K. F. Laserson, A. Mohammed and P. A. Phillips-Howard (2013). "'We keep it secret so no one should know'--a qualitative study to explore young schoolgirls attitudes and experiences with menstruation in rural western Kenya." PLoS ONE **8**(11): e79132.

Mishra, S.K., D. Dasgupta and S. Ray (2017). "A study on the relationship of sociocultural characteristics, menstrual hygiene practices and gynaecological problems among adolescent girls in Eastern India." Int J Adolesc Med Health **29**(5): 20150111.

Montgomery, P., J. Hennegan, C. Dolan, M. Wu, L. Steinfield and L. Scott (2016). "Menstruation and the Cycle of Poverty: A Cluster Quasi-Randomised Control Trial of Sanitary Pad and Puberty Education Provision in Uganda." PLoS ONE **11**(12): e0166122.

Moodi, M., N. Zamanipour, G.-R. Sharifirad and H. Shahnazi (2013). "Evaluating puberty health program effect on knowledge increase among female intermediate and high school students in Birjand, Iran." Journal of Education and Health Promotion **2**: 57.

Mumtaz, S. and S. K. Ansari (2016). "Psychology of Beliefs and Practices Relating to Menstrual Hygiene of Adolescent Girls in Rural, Islamabad, Pakistan." Imperial Journal of Interdisciplinary Research **2**(7): 670-677.

Nagaraj, C. and K. S. Konapur (2016). "Effect of health Education on Awareness and Practices related to menstruation among rural adolescent school girls in Bengaluru, Karnataka." International Journal **2**(1): 19.

Phillips-Howard, P. A., E. Nyothach, F. O. Ter Kuile, J. Omoto, D. Wang, C. Zeh, C. Onyango, L. Mason, K. T. Alexander, F. O. Odhiambo, A. Eleveld, A. Mohammed, A. M. van Eijk, R. T. Edwards, J. Vulule, B. Faragher and K. F. Laserson (2016). "Menstrual cups and sanitary pads to reduce school attrition, and sexually transmitted and reproductive tract infections: a cluster randomised controlled feasibility study in rural Western Kenya." BMJ Open **6**(11): e013229.

Pratibha, S., A. S. Nawaz and A. Kumar (2016). "Impact of Menstrual Practices on the Health of Adolescent Girls and the Challenges Faced in Menstrual Hygiene Management at Schools." National Journal of Community Medicine **7**(8): 667-671.

Ramachandra, K., S. Gilyaru, A. Eregowda and S. Yathiraja (2016). "A study on knowledge and practices regarding menstrual hygiene among urban adolescent girls." International Journal of Contemporary Pediatrics **3**(1): 142-145.

Saghi, S., M. Mirghafourvand, S. M. Alizadeh Charandabi, A. Nabighadim, S. Seidi and A. Rahmani (2016). "Knowledge and attitude about pubertal health and their socio-demographic predictors in Iranian adolescents." Int J Adolesc Med Health **28**(4): 397-405.

Shah, S. P., R. Nair, P. P. Shah, D. K. Modi, S. A. Desai and L. Desai (2013). "Improving quality of life with new menstrual hygiene practices among adolescent tribal girls in rural Gujarat, India." Reprod Health Matters **21**(41): 205-213.

Sharma, I. and E. Moktan (2017). "Assessing the level of Knowledge and Practice of Menstrual Hygiene among Adolescent School girls in Siraha district of Nepal: A cross sectional study." Asian Journal of Multidisciplinary Studies **5**(10).

Sharma, R., S. Negi, D. Kunj, V. Sharma and Vardha (2015). "Menstrual hygiene among adolescent girls." Indian Journal of Community Health **27**(3): 376-380.

Sharma, S., S. Choudhary, N. Saluja, D. Gaur, S. Kumari, S. Pandey and H. Hisar (2016). "Knowledge and practices about menstrual hygiene among school adolescent girls in Agroha village of Haryana." Age **64**: 42.47.

Shoor, P. (2017). "A study of knowledge, attitude, and practices of menstrual health among adolescent school girls in urban field practice area of medical college, Tumkur." Indian Journal of Health Sciences and Biomedical Research (KLEU) **10**(3): 249.

Sridhar, D. and N. Gauthami (2017). "Menstrual health status and cultural practices of tribal adolescent girls." International Journal Of Community Medicine And Public Health **4**(11): 4120-4124.

Su, J. J. and D. Lindell (2016). "Promoting the menstrual health of adolescent girls in China." Nurs Health Sci **18**(4): 481-487.

Um, L., N. W. Yusuf and A. B. Musa (2010). "Menstruation and Menstrual Hygiene amongst Adolescent School Girls in Kano, Northwestern Nigeria." African Journal of Reproductive Health / La Revue Africaine de la Santé Reproductive **14**(3): 201-207.

Valizadeh, S., M. Assdollahi, M. Mirghafourvand and A. Afsari (2017). "Educating Mothers and Girls about Knowledge and Practices toward Puberty Hygiene in Tabriz, Iran: A Randomized Controlled Clinical Trial." Iranian Red Crescent Medical Journal **19**(2): 10.
